# Gaillard’s Medical Journal

**Published:** 1890-02

**Authors:** 


GAILLARD'S MEDICAL JOURNAL, one of the best American monthly medical magazines, in its January number, 1890, thus gracefully speaks in its editorial columns: "In the February number we will publish with illustrations the valuable paper (read before the Southern Surgical and Gynecological Association) of Dr. L. S. McMurtry, of Danville, Ky., one of the most accomplished physicians and cultivated gentlemen in the American profession."

---

MUNDÉ says that to the imprudent act of getting out of bed without protecting the feet—one so commonly committed by women without thought of the consequences—may be traced many an attack of cellulitis, brought on by the sudden though momentary exposure of the feet to cold. It has caused more diseases to women, previously healthy, than could result from any other single act of imprudence.—*Lancet-Clinic.*

